# A human Angelman Syndrome class II pluripotent stem cell line with fluorescent paternal *UBE3A* reporter

**DOI:** 10.3389/fcell.2025.1665693

**Published:** 2025-08-29

**Authors:** Gautami R. Kelkar, Samantha R. Stuppy, Dilara Sen, Z. Begum Yagci, Linna Han, Lexi Land, Jessica K. Hartman, Albert J. Keung

**Affiliations:** ^1^ Department of Chemical and Biomolecular Engineering, North Carolina State University, Raleigh, NC, United States; ^2^ Genetics and Genomics Academy, North Carolina State University, Raleigh, NC, United States; ^3^ Cell Microsystems, Durham, NC, United States

**Keywords:** Angelman Syndrome, hiPSC, *UBE3A* reactivation, fluorescent reporter, cerebral organoids, neurodevelopment, therapeutic screening, imprinting

## Abstract

**Introduction:**

Angelman Syndrome (AS) is characterized in large part by the loss of functional UBE3A protein in mature neurons. A majority of AS etiologies is linked to deletion of the maternal copy of the *UBE3A* gene and epigenetic silencing of the paternal copy. A common therapeutic strategy is to unsilence the intact paternal copy thereby restoring UBE3A levels. Identifying novel therapies has been aided by a *UBE3A-YFP* reporter mouse model. This study presents an analogous fluorescent *UBE3A* reporter system in human cells.

**Methods:**

Previously derived induced Pluripotent Stem Cells (iPSCs) with a Class II large deletion at the *UBE3A* locus are used in this study. *mGL* and *eGFP* are integrated downstream of the endogenous *UBE3A* using CRISPR/Cas9. These reporter iPSCs are differentiated into 2D and 3D neural cultures to monitor long-term neuronal maturation. Green fluorescence dynamics are analyzed by immunostaining and flow cytometry.

**Results:**

The reporter is successfully integrated into the genome and reports paternal *UBE3A* expression. Fluorescence expression gradually reduces with *UBE3A* silencing in neurons as they mature. Expression patterns also reflect expected responses to molecules known to reactivate paternal *UBE3A*.

**Discussion:**

This human-cell-based model can be used to screen novel therapeutic candidates, facilitate tracking of *UBE3A* expression in time and space, and study human-specific responses. However, its ability to restore UBE3A function cannot be studied using this model. Further research in human cells is needed to engineer systems with functional UBE3A to fully capture the therapeutic capabilities of novel candidates.

## 1 Introduction

*UBE3A* encodes an E3 ubiquitin ligase (UBE3A, E6-AP) that is integral for several cellular processes that govern cortical development (LaSalle et al., 2015; Khatri and Man, 2019). While it is biallelically expressed in most early developmental cell types, *UBE3A* switches to monoallelic expression in mature neurons (Albrecht et al., 1997; Rougeulle et al., 1997; Vu and Hoffman, 1997). The paternal allele is epigenetically silenced by a long non-coding RNA transcript, *UBE3A-ATS*, leaving only the maternal allele actively transcribed. Loss or loss of function of the maternal allele leads to a dearth of UBE3A and is the primary linkage to the neurodevelopmental disorder, Angelman Syndrome (AS) (Angelman, 1965; Kishino et al., 1997; Matsuura et al., 1997; Sutcliffe et al., 1997).

The predominant etiologies affecting maternal *UBE3A* are large genomic deletions comprising approximately 70% of all AS (Keute et al., 2021). Since the paternal *UBE3A* allele is still intact, therapeutic strategies often target its unsilencing via inhibition or knockdown of *UBE3A-ATS*. Thus, the ability to track the transcriptional activity of paternal *UBE3A* would facilitate therapeutic screening. It would also benefit the spatiotemporal mapping of allele-specific *UBE3A* expression, which is a highly dynamic and cell-type-specific process (Burette et al., 2018; 2017; Estridge et al., 2025; Judson et al., 2014; Sen et al., 2021; 2020). The *UBE3A-YFP* reporter mouse model (Dindot et al., 2008) has been leveraged to screen for therapeutic molecules, identifying topoisomerase inhibitors (Huang et al., 2012; H.-M. Lee et al., 2018), anti-sense oligonucleotides (ASOs) (Clarke et al., 2024; Lee et al., 2023; Meng et al., 2015), CRISPR-Cas9 gene therapies (Bazick et al., 2024; Schmid et al., 2021; Wolter et al., 2020), and small molecules like PHA533533 (Vihma et al., 2024). Future screens, preclinical validations, and research studies would all benefit from an analogous human system (Sen et al., 2021). It would account for species-specific differences in (epi)genetic variation, developmental timescales, dose-response characteristics, and toxicities (Chen, 2016). In addition, such models could benefit mechanistic studies on *UBE3A* dynamics and its role in cellular processes (Condon et al., 2013; Filonova et al., 2014; Grier et al., 2015; Hillman et al., 2017; Jones et al., 2016; Judson et al., 2014; Kaphzan and Hernandez, 2012; McCoy et al., 2017; Sonzogni et al., 2020). In a few cases, compound hits from the mouse model were then validated on human iPSC-derived neurons, but these cellular systems required the use of antibody labeling or amplification which need laborious fixation steps and have known challenges of sensitivity and specificity (Dindot et al., 2023; Sen et al., 2020).

Here, we modify a human AS Class II patient-derived induced pluripotent stem cell (iPSC) line by knocking in a fluorescent reporter in frame with paternal *UBE3A* (Chamberlain et al., 2010). These iPSCs are differentiated into 2D neurons as well as cerebral organoids to observe changes in fluorescence levels with time, in specific cell types, and with neural cell maturation (Ciceri et al., 2024; Lancaster et al., 2013). We also assess *UBE3A* reactivation upon exposure to topoisomerase inhibitors.

## 2 Materials and methods

### 2.1 DNA plasmids

Two donor plasmids expressing GSLinker-eGFP and 2A-eGFP were generated. However, the fluorescence signal was not successfully detected in iPSCs. Hence, the final *UBE3A*-targeting construct was designed for both direct and indirect expression: UBE3A-GSLinker-mGreenLantern-IRES-NLS-eGFP. First, GSLinker-eGFP (pDS44-GSLinker) was subcloned into the CIRTS-1: ORF5-TBP6.7-Pin domain-NLS plasmid (Addgene #132543) by restriction enzyme digestion using NdeI and MfeI, followed by an overnight T4 ligation reaction at 16 °C. The resulting plasmid (pSS12-GSLinker-eGFP) was digested using BseRI and BstEII to remove the GS linker and to perform Gibson assembly to add PAM mutations and an IRES-NLS sequence. Finally, a GSLinker-mGreenLantern fragment was added before the IRES via restriction digestion at BstEII and AvrII sites followed by Gibson assembly to obtain pSS23-GSLinker-mGreenLantern-IRES-NLS-eGFP. Both Gibson assembly reactions were performed at 50 °C for 1 h with 100 ng of the backbone and 2-fold molar excess of the insert. For the Cas9-gRNA construct, a modified pX330-U6-Chimeric_BB-Cbh-hSpCas9 (Addgene #42230) was digested using Bbs1, along with rSAP (NEB) for dephosphorylation of the backbone. The gRNA (AGGCCATCACGTATGCCAA (C. Sirois, 2018)) was cloned into the linearized backbone. All restriction digestion enzymes were procured from NEB, and reactions were performed at 37 °C for 1 h according to the manufacturer’s protocol. The donor construct pSS22-IRES- NLS-eGFP along with information about the GSLinker-mGreenLantern fragment (# 241837) and Cas9-gRNA construct pDS48 (# 241839) used in the present study are available from Addgene.

### 2.2 Cell transfection and genomic PCR screening

AS Class II deletion iPSCs (developed by Chamberlain and colleagues, and procured from Kerafast) were transfected via electroporation (Chamberlain et al., 2010). The experiment used 1.5 μg of pSS23 and 1.5 μg of pDS48 in a 10 μL Neon^™^ Transfection System (Invitrogen) reaction containing 50,000 cells. The following conditions were used: 950 V - 2 pulse - 30 width. Cells were immediately seeded post-transfection onto a growth factor-reduced Matrigel-coated (Corning) CellRaft AIR® array from Cell Microsystems and allowed to recover for about a week with gentle mTeSR Plus (StemCell Technologies) media changes. Upon expansion, the cells were dissociated with Accutase (BioLegend) and seeded into 96-well plates (Corning COSTAR^™^). Replicate plates were generated and screened by PCR of genomic DNA. All media contained CloneR (StemCell Technologies) per the manufacturer’s instructions from transfection through expansion and re-plating. Genomic PCR screens were performed with primer sets SRS21 + SRS24, followed by Sanger sequencing to identify a polyclonal population. These cells were further sorted using the CellRaft AIR® system, and the aforementioned PCR screening was repeated. An additional PCR screening step was performed with SRS21 + DSP206 primers, followed by Nanopore sequencing (performed by SNPsaurus) to identify 3 monoclonal populations of reporter iPSCs. The sequences for primers can be found in Supplementary Table S1.

### 2.3 Human pluripotent stem cell (hPSC) culture

The parental AS Class II deletion iPSCs, the reporter iPSCs, and the H9_*UBE3A*__*m-/p-*_ ESCs (from Dr. Stormy Chamberlain) were maintained on growth factor-reduced Matrigel (Corning) coated 6-well plates (Corning COSTAR^™^, Fisher Scientific) in mTeSR Plus (StemCell Technologies). Cells were passaged every 3–5 days as necessary using 0.5 mM EDTA (Invitrogen).

### 2.4 RNA extraction and qPCR

Parental and reporter iPSCs were washed with cold 1X PBS (Gibco), and total RNA was isolated using the RNeasy® Mini Kit (Qiagen) following manufacturer’s instructions. RNA samples were treated with the Turbo DNA-free^™^ kit (Invitrogen) to remove DNA contamination. Reverse transcription was performed using the iScript Advanced cDNA Synthesis Kit (BIO-RAD) according to the manufacturer’s protocol. qPCR reactions were performed using the SsoAdvanced SYBR Green Supermix (BIO-RAD) according to the manufacturer’s protocol. Primers SRS35 and SRS36 were used to amplify 2 regions: one within *mGL* and *eGFP* each, and primers SRS37 and SRS38 were used to amplify a region within *eGFP* only. *GAPDH* was used as the reference gene. For Supplementary Figure S6, RNA was extracted and purified using the Direct-zol RNA Miniprep from Zymo Research following the manufacturer’s protocol. Reverse transcription was performed as mentioned above. cDNA was incubated with TaqMan Master Mix and probes for *UBE3A-ATS* (Hs01372957_m1) and the reference gene *PPIA* (Hs99999904_m1) according to the manufacturer’s (ThermoFisher Scientific) instructions. Data was analyzed in MS Excel and is presented as ∆Ct relative to the reference gene. Note that the Ct values for the target genes are greater than those for the reference genes, so smaller ∆Ct magnitudes correspond to greater expression levels. The sequences for primers can be found in Supplementary Table S1.

### 2.5 2D neural culture

All media for neural cultures were sourced from StemCell Technologies, and the procedures adhered to their established protocols. iPSCs were differentiated to Neural Progenitor Cells (NPCs) using the StemDiff^™^ SMADi neural induction kit and stored in liquid nitrogen. Frozen NPCs were thawed and grown to confluency before being differentiated into neural precursor cells. Confluent NPCs were passed using Accutase (BioLegend) to a well of a 6-well plate (Corning COSTAR^™^, Fisher Scientific) at 1.3 × 10^5^ cells/cm^2^ using the StemDiff^™^ Forebrain Neuron Differentiation Kit. Upon reaching confluency, these cells were passed using Accutase into 8-well chamber slides (Falcon) at 2.00 × 10^4^ cells/cm^2^ and maintained using the StemDiff^™^ Forebrain Neuron Maturation Kit. The media was changed every 2–3 days. To accelerate neuron maturation, 4 μM GSK343 (MilliporeSigma) was added at every media change (Ciceri et al., 2024).

### 2.6 Cerebral organoid culture

Whole-brain organoids were generated from AS Class II deletion parental and reporter stem cell lines. The compositions of the media described here are in accordance with the Lancaster Whole Brain protocol (Lancaster et al., 2013). hiPSCs were allowed to reach 80%–90% confluency before they were dissociated into a single-cell suspension using Accutase (BioLegend) and re-plated in low-adhesion U-bottom 96-well plates (Corning COSTAR^™^, Fisher Scientific) at 12,000-15,000 cells/well in hPSC growth medium (∼v/v 76% DMEM/F12 (Gibco), 19% KnockOut Serum Replacement (Gibco), 3% Fetal Bovine Serum (Avantor), 1% GlutaMAX (Gibco), 1% MEM-Non-Essential Amino Acids (Cytiva), and 100 μM 2-mercaptoethanol (Amresco)) supplemented with 50 μM Y-27632 (StemCell Technologies) and 4 ng/mL bFGF (Invitrogen). Partial media changes were performed every 2 days until Day 6, and bFGF was removed on Day 4. On Day 6, organoids were switched to neural induction medium (∼v/v 97% DMEM/F12, 1% GlutaMAX, 1% MEM-Non-Essential Amino Acids, 1% N2 Supplement (Gibco), and 1 μg/mL Heparin (Sigma Aldrich)) by a complete media change. Partial media changes were performed every 2 days. On Day 11, organoids were embedded in 35 μL Geltrex^™^ (Gibco) and transferred to 10 cm plates (Fisher Scientific) in Cerebral Organoid Differentiation (COD) medium without vitamin A (∼v/v 48% DMEM/F12, 48% Neurobasal medium (Gibco), 1% GlutaMAX, 0.5% MEM- Non-Essential Amino Acids, 0.5% N2 Supplement, 1% 100X Penicillin/Streptomycin (Genesee), 1% B27 Supplement without vitamin A (Gibco), 50 μM 2-mercaptoethanol, and 2.5 μg/mL Insulin (Gibco)), which was switched to COD medium with vitamin A (substituting 1% B27 Supplement with B27 Supplement containing vitamin A (Gibco)) on Day 15, and the plates were transferred to an orbital shaker at 70 RPM. To accelerate neuron maturation, 4 μM GSK343 (MilliporeSigma) was added to the organoids between Days 17–25, with partial media changes carried out every other day (Ciceri et al., 2024). The organoids remained in COD media with vitamin A for up to 17 weeks with media changes every 3–4 days after Day 25.

### 2.7 Histology and immunofluorescence

**FIGURE 1:**
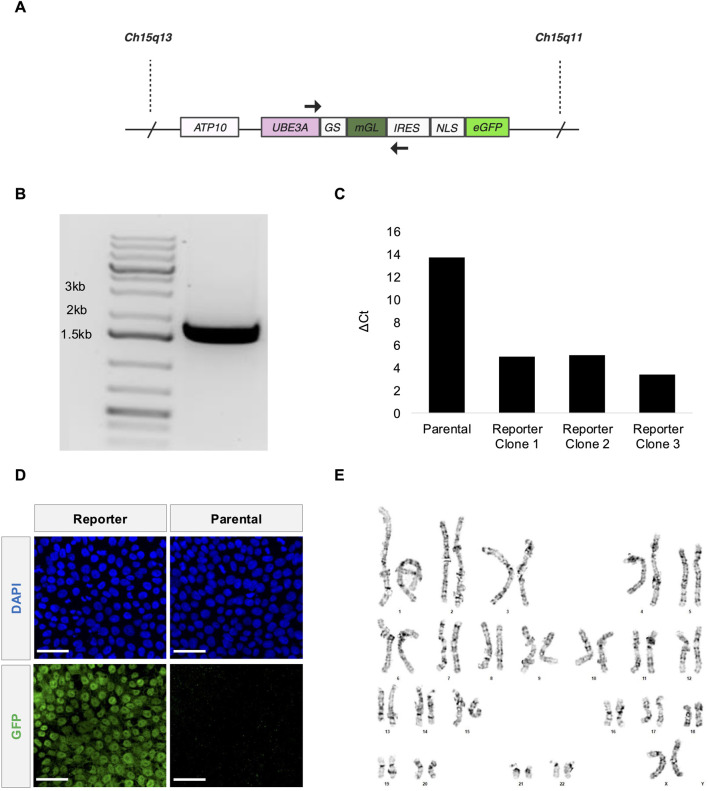
Fluorescent *UBE3A* reporter knocked into AS Class II deletion iPSCs. **(A)** Illustration of the *UBE3A* locus in paternal Chromosome 15 after reporter integration. **(B)** Primers SRS21 (forward) and SRS24 (reverse) targeting the *UBE3A*-reporter region shown in 1A amplified a band that is ∼1.756 kb from genomic DNA of the polyclonal reporter cells. **(C)** qRT-PCR measurements of fluorescent reporter mRNA (Supplementary Figure S1A). ∆Ct values relative to GAPDH presented for the parental iPSCs and the three monoclonal reporter iPSCs. **(D)** Representative confocal microscopy images showing GFP expression in the nuclei (DAPI) of AS Class II deletion parental iPSCs (right) and the edited reporter iPSCs (left). Scale bars are 50 µm. **(E)** G-banded chromosomes show normal chromosome counts of 46 XX for reporter stem cells at passage number 5 post editing. The karyotype analysis was performed by LabCorp. Editing was performed on the parental stem cell line at passage number 39.

**FIGURE 2:**
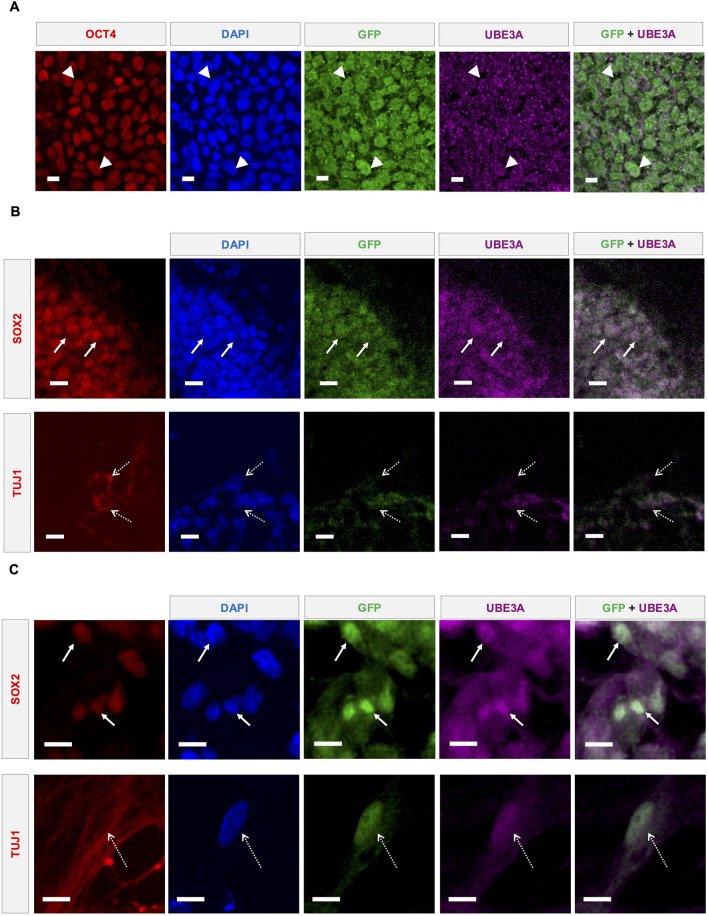
Direct UBE3A labeling and reporter fluorescence coexpress in multiple cell types. **(A)** Representative confocal microscopy images showing UBE3A and GFP expression in the nuclei (DAPI) of reporter iPSCs. Costes-corrected Mander’s coefficients: Reporter → UBE3A = 0.78; UBE3A → Reporter = 0.85. OCT4 was used as a marker to identify iPSCs. Representative confocal microscopy images showing UBE3A and GFP expression in the nuclei (DAPI) of cells in edited reporter iPSC-derived **(B)** cerebral organoids and **(C)** 2D neural cultures. SOX2+ neural precursors shown using solid arrows and TUJ1+ neurons marked by dashed arrows. All scale bars are 10 µm.

**FIGURE 3:**
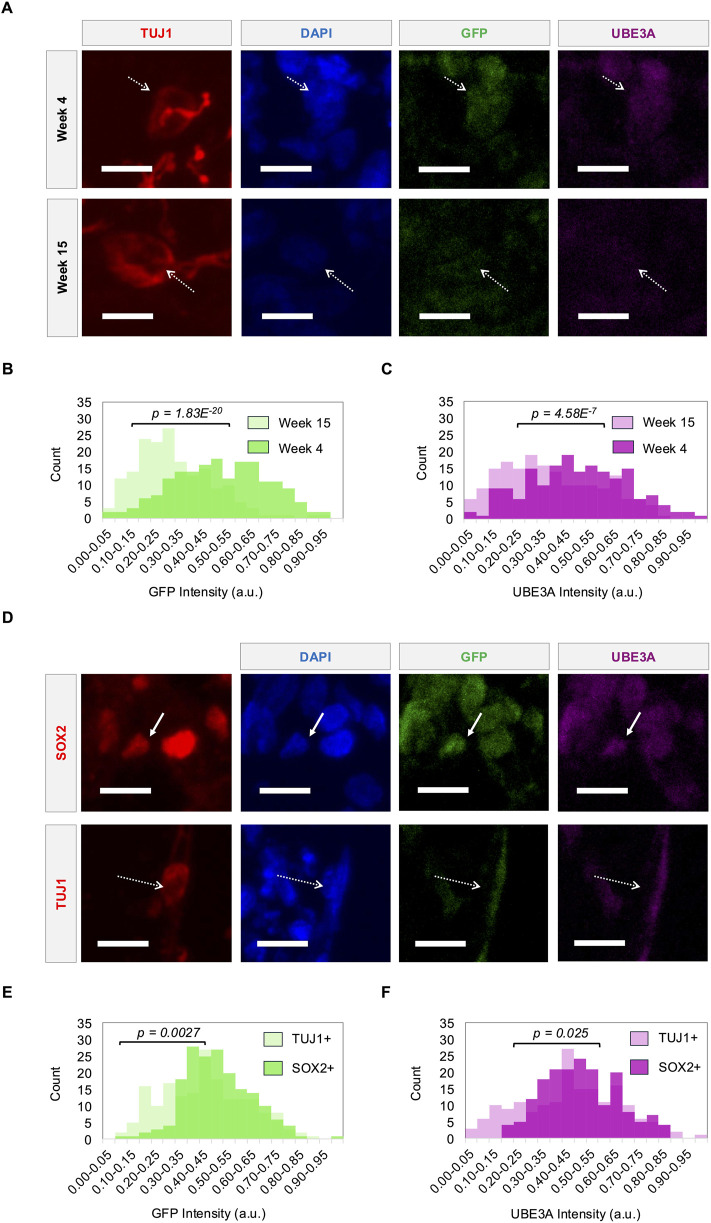
Reporter fluorescence tracks UBE3A expression dynamics during neural differentiation. **(A)** Representative images showing UBE3A and GFP expression in the nuclei (DAPI) of TUJ1+ neurons (dashed arrows) in cerebral organoids at week 4 and week 15. **(B)** GFP and **(C)** UBE3A intensities of nuclei in TUJ1+ neurons at weeks 4 and 15. **(D)** Representative images showing UBE3A and GFP expression in the nuclei of SOX2+ precursors (solid arrows) and TUJ1+ neurons (dashed arrows) in cerebral organoids at week 9. **(E)** GFP and **(F)** UBE3A intensities of nuclei in TUJ1+ neurons and SOX2+ precursors at week 9. Populations were compared using a two-sample t-test, n = 180 cells (3 organoids). All scale bars are 10 μm. a.u. - arbitrary units.

Tissues were fixed in 4% paraformaldehyde for 20 min at 4 °C followed by washing in 1X PBS (Gibco) three times for 10 min. Tissues were allowed to equilibrate in 30% sucrose overnight and then embedded in 10%/7.5% sucrose/gelatin. Embedded tissues were frozen in an isopentane and dry ice bath at −30 to −50 °C and stored at −80 °C. Prior to analysis, they were cryosectioned into 30 μm slices using a cryoStat (ThermoFisher). iPSCs and 2D neural cultures plated in 8-well chamber slides (Falcon) were fixed in 4% paraformaldehyde for 10 min at room temperature followed by washing in 1X PBS (Gibco) three times for 5 min. For immunohistochemistry, organoid sections were blocked and permeabilized in 1% Triton X-100 and 5% normal donkey serum (Jackson Immunoresearch Labs) in 1X PBS. For 2D cultures, 1% normal donkey serum in 1% Triton X-100 was used instead. Both 2D cultures and organoid sections were then incubated with primary antibodies in 10% Triton X-100, 1% normal donkey serum in UltraPure^™^ water (Invitrogen) and 10X PBS overnight at 4 °C at the following dilutions: UBE3A (rabbit, Bethyl Laboratories, 1:250 or mouse, MilliporeSigma, 1:1000 (Figure 2A)), GFP (chicken, abcam, 1:2000 for iPSCs and 2D neural cultures; 1:3000 for organoid sections), OCT4 (rabbit, Cell Signaling, 1:200), SOX2 (goat, R&D Systems, 1:200), and TUJ1 (mouse, MilliporeSigma, 1:500). A300-351A antibody from Bethyl Laboratories and SAB1404508 from Millipore Sigma were chosen based on a previous study comparing commercially available UBE3A antibodies (Sen et al., 2021). Following primary antibodies, sections were incubated with secondary antibodies - donkey Alexa Fluor 488, 546, and 647 conjugates (Invitrogen, 1:250) in 10% Triton X-100, 1% normal donkey serum in UltraPure^™^ water (Invitrogen) and 10X PBS for 2 h at room temperature, and the nuclei were stained with DAPI (Invitrogen). Slides were mounted using ProLong^™^ Antifade Diamond (Invitrogen). Images were taken with Nikon A1R confocal microscope (Nikon Instruments). Images in Figures 1D, 2A,C, and Supplementary Figures S3 and S7 were captured using a CFI plan Apochromatic 10X air objective, and images in Figures 2B, 3A,D were captured using a S Plan Fluor ELWD 20X air objective. All images were acquired using a Galvano scanner on a Nikon A1R confocal microscope with the following laser lines: 405 nm (DAPI), 488 nm (GFP, OCT4 for Supplementary Figure S3), 561 nm (SOX2, TUJ1, OCT4), and 640 nm (UBE3A), and a gallium arsenide phosphide (GaAsP) PMT detector. Images in Figures 1D, 2A,C, 3A,D, and Supplementary Figures S3, S7 were captured at a resolution of 1024 × 1024 pixels and images in Figure 2B were captured at 2048 × 2048-pixel resolution. Images in Figure 2A; Supplementary Figure S3 were captured as 2 μm z-stacks, Supplementary Figure S7 were captured as 2.5 μm z-stacks, Figures 2B, 3A,D, were captured as 3 μm z-stacks, and Figure 2C were captured as 4 μm z-stacks.

### 2.8 Image analysis and quantification

All samples within experiments were imaged using the same microscope settings and z-stack step sizes. For Figure 1D, a CellProfiler-automated pipeline was used to identify nuclei (DAPI) and measure integrated GFP intensity within these regions. The GFP intensity for each nucleus in all images was normalized to the corresponding DAPI intensity. For Figures 1, 3; Supplementary Figures S3, S7. S8, a maximum intensity projection image was created from z-stacks, background was subtracted, and intensities for all channels were adjusted identically in FIJI (Schindelin et al., 2012) within each experiment. For colocalization quantification in Figure 2A, Costes-corrected Mander’s correlation coefficient (GFP ↔ UBE3A), was calculated using the MeasureColocalization module in CellProfiler for the entire image. For UBE3A and GFP intensity measurements over time in Figure 3, three organoids per time-point, and three regions per organoid were imaged. Within each image, 20 ROIs were manually selected for each cell type (180 cells per condition) in FIJI as follows: for neural precursor cells, DAPI + regions that showed overlapping signal with SOX2, and for neurons, DAPI + regions that were enclosed by TUJ1 signals. Mean Intensities for the selected ROIs were measured for GFP and UBE3A channels after identical LUT adjustment for all images. Identical normalization was applied to each image where intensities for each channel within each image were normalized to a range of 0–1 as follows:

$$\text{Normalized intensity for each nucleus}=\frac{\text{Absolute}\;\text{intensity}\;‐\;\text{Background}}{\mathrm{Max}\;\text{intensity}\;\text{nucleus}\;‐\;\text{Background}}$$

For Supplementary Figure S7B, a CellProfiler-automated pipeline was used to identify nuclei (DAPI) and measure integrated GFP intensity within these regions. The CellProfiler pipelines and parameters are now included in the Supplementary Information. For Supplementary Figure S8, three organoids per condition, two regions per organoid were imaged. ROI selection (10-15 per image), GFP and UBE3A intensity measurement, and normalization methods were the same as above for Figure 3.

### 2.9 Topoisomerase inhibitor treatment

Topotecan hydrochloride and irinotecan hydrochloride (both from Molcan Corporation) were directly added to 17-week-old AS reporter organoids at a final concentration of 1 μM in culture. 0.2% DMSO in water was used as the vehicle control. Organoids were incubated at 37 °C on an orbital shaker at 90 RPM for 72 h before being harvested for analysis.

### 2.10 Flow cytometry and data visualization

Adherent iPSCs were washed with 1 mL 1X PBS (Gibco), incubated with Accutase (BioLegend) for 5 min at 37 °C, and dissociated into a single-cell suspension. Organoids were also washed with 1X PBS, incubated in Accutase for 3 rounds of 10 min each at 37 °C with pipetting to break up the organoids between rounds, ultimately obtaining a single-cell suspension. For both iPSCs and organoids, the single-cell suspensions were centrifuged at 300 *g* for 5 min, Accutase was removed, and the cells were re-suspended in 300 nM DAPI (Invitrogen) in 1X PBS. The samples were then run on the flow cytometer (MACSQuant® VYB), at an average flow rate of 50 μL/min with identical volumes of 450 µL for all samples within an experiment. Green fluorescence was measured using the 488 nm laser and the B1 channel (525/50 nm emission filter), with a photomultiplier tube (PMT) gain set to 285 V. The data was processed and visualized using FlowJo^™^ v10.10.0 software (BD Life Sciences). For each sample, viable cell populations were selected from an identical region on the SSC-A vs. FSC-A plot. Single-cells were selected from this population using the FSC-H vs. FSC-A plot. Green fluorescence intensity histograms were derived from these single viable cells with event count maintained at 444,565 across all samples. Parental stem cells, which lack the fluorescent reporter, were used as the negative control to account for cellular autofluorescence, and edited stem cells were used as the positive control for green fluorescent reporter expression to identify the green fluorescence gates.

### 2.11 UBE3A ubiquitin ligase activity assay

The assay followed the protocol described in (Han et al., 2025). Briefly, lysates from all 3 cell lines (3 biological replicates per cell line) were collected using the Pierce^™^ IP lysis buffer (Fisher Scientific). The total amount of protein present in the cell lysates was measured using Pierce^™^ BCA Protein Assay Kit (ThermoFisher Scientific) following which the lysates were concentrated using Amicon® ultra centrifugal filters (MilliporeSigma, 50 kDa MWCO, 0.5 mL). The concentrated lysate was used in the ubiquitin ligase activity reaction mixture. The reaction components and their approximate working concentrations/amounts were as follows: 10X reaction buffer (50 mM HEPES, 50 mM NaCl), Mg^2+^/ATP (10 mM, Enzo Life Sciences), UBE1 (100 nM, R&D Systems), UBE2 (1 μM, R&D Systems), HPV-E6 (1.5 µM, R&D Systems), custom p53 substrate (5.4 µg, Addgene plasmid # 233738) and ubiquitin-fluorescein (10 μM, UBP Bio). Amicon® filter-concentrated IP lysis buffer was used as the blank control. The samples were incubated at 37 °C for an hour. HisPur^™^ Ni-NTA magnetic beads (Fisher) were used to pull down p53 substrates. To detect ubiquitin-fluorescein binding, end-point fluorescence of the p53 substrates was measured using a TECAN Infinite 200 Pro microplate reader with 485 nm excitation and 535 nm emission filters. To calculate the normalized fluorescence readings, the fluorescence reading of the IP lysis buffer control was first subtracted from the readings of the samples, and then these readings were normalized to their corresponding approximate protein amounts.

### 2.12 Data presentation and statistical analysis

All statistical analyses were performed in MS Excel. For Figures 3B,C,E,F; Supplementary Figures S3, S4, normalized intensity values from 180 cells are presented as a binned histogram. A two-sample t-test assuming unequal variances was used for statistical comparison of the two conditions in each plot. For Figure 6, each bar represents the average FITC intensity (a.u.) of 3 biological replicates, with error bars showing 95% confidence intervals. For statistical analysis, one-way ANOVA was performed across the 3 conditions, followed by a Tukey-Kramer *post hoc* test for 1-1 comparisons. For Supplementary Figure S1C, bars represent average normalized intensities from 1560 (parental) and 1328 (reporter) nuclei, with error bars showing 95% confidence intervals. The conditions were compared by a two-sample t-test assuming unequal variances. For Supplementary Figure S6, each bar represents the average ΔCt of 3 biological replicates, with error bars showing 95% confidence intervals. The conditions were compared by a two-sample t-test assuming unequal variances. p-values from all statistical tests are presented on the plots. For Supplementary Figure S7B, bars represent average intensity from 1646 nuclei for both cell lines, with error bars showing 95% confidence intervals. The conditions were compared by a two-sample t-test assuming unequal variances. For Supplementary Figure S8, normalized intensity values from 160 to 170 cells are presented as binned histograms.

## 3 Results

### 3.1 Fluorescent *UBE3A* reporter knocked into AS Class II deletion iPSCs

Here we edit an AS Class II iPSC line harboring a deletion of 15q11-q13 on the maternal chromosome (Chamberlain et al., 2010), with three different constructs. All three constructs target the C-terminus of paternal *UBE3A* using CRISPR/Cas9-induced homology directed repair. These include in frame insertions prior to the *UBE3A* stop codon of: *GSLinker-eGFP*; *2A-eGFP*; and *GSLinker-mGreenLantern (mGL)-IRES-NLS-eGFP*. The first construct, if successful, would have provided the ability to directly track the localization of UBE3A. With the potential of direct fusions altering UBE3A activity (and UBE3A localization when fused to the N-terminus), the second construct would translate eGFP as a separate protein, providing an indirect readout of UBE3A levels as it is not directly fused to UBE3A. The third construct was designed to increase the signal due to the expression of two fluorophores. It remains unclear why only the third construct (Figure 1A) yielded a detectable signal. It is possible the first two constructs adversely affected native splicing as there are complicated intronic/exonic structures near the end of the coding region. Genotyping indicates this construct is successfully integrated into the genome (Figure 1B). The Cell Microsystems’ CellRaft AIR® system enables isogenic clonal populations to be further isolated. Expression of the reporter transcript is confirmed using qRT-PCR (Figure 1C; Supplementary Figure S1A, B) for all three clonal populations. One clone is selected for further analysis. At the protein level, the reporter iPSCs show higher antibody-enhanced green fluorescence compared to the parental iPSCs in confocal imaging (Figure 1D; Supplementary Figure S1C). The reporter cell line maintains a normal karyotype (Figure 1E; Supplementary Figure S2) and pluripotency (Supplementary Figure S3).

### 3.2 Direct UBE3A labeling and reporter fluorescence coexpress in multiple cell types

The coexpression of fluorescent reporters with UBE3A is essential for their utility. Antibody-enhanced fluorescence imaging indicates coexpression of the fluorescent proteins with UBE3A (Figure 2A), albeit with the UBE3A signal being relatively noisier than the reporter. In AS deletion etiologies, paternal *UBE3A* is known to be expressed in pluripotent stem cells, neural precursors, and immature neurons of murine (Judson et al., 2014) and human (Chamberlain et al., 2010; Sen et al., 2020) models. As expected based on this prior literature, coexpression is also observed in SOX2+ neural precursors and TUJ1+ neurons of 4-week-old cerebral organoids generated from the reporter iPSCs (Figure 2B). Similarly, in 9-week-old 2D neural cultures, expression is observed in both SOX2+ and TUJ1+ cells (Figure 2C). The nuclear localization of UBE3A in neurons, and even the cytoplasmic and nuclear distribution in other cell types, is consistent with previous reports examining neurodevelopmental cell types in human iPSC-derived models of AS (Zampeta et al. 2020; Munshi 2019; Sen et al. 2020), confirming that the C-terminal reporter fusion does not alter the localization dynamics of UBE3A.

### 3.3 Reporter fluorescence tracks UBE3A expression dynamics during neural differentiation

During neurodevelopment, paternal *UBE3A* is gradually silenced over time during neuronal differentiation and maturation. Fluorescence imaging of long-term cerebral organoid cultures at weeks 4, 9, 12, and 15 show that the reporter tracks this silencing. Consistent with prior findings, UBE3A exhibits strong nuclear localization up to week 12, which becomes weaker and more diffuse by week 15 (Sen et al., 2020) (Figure 3A). Reporter expression mirrors this pattern. Quantitative analysis reveals a significant decline in nuclear fluorescence intensities of both UBE3A and the reporter by week 12 (Supplementary Figure S4), and this is sustained through week 15 (Figures 3B,C). Furthermore, silencing dynamics also track cellular differentiation where TUJ1+ neurons consistently show weaker nuclear UBE3A and reporter signals compared to their signals within SOX2+ neural precursors (Figure 3D). Notably, this difference is evident as early as week 9, with neurons displaying significantly lower intensities than precursors (Figures 3E,F; Supplementary Figure S5). This decrease in UBE3A and reporter protein levels is corroborated by increased *UBE3A-ATS* transcript expression in 17-week-old reporter organoids compared to the reporter iPSCs (Supplementary Figure S6). These results confirm the silencing of paternal *UBE3A* during neural maturation and demonstrate that the reporter faithfully tracks this dynamic expression.

### 3.4 Unamplified native reporter fluorescence is detectable by flow cytometry

**FIGURE 4:**
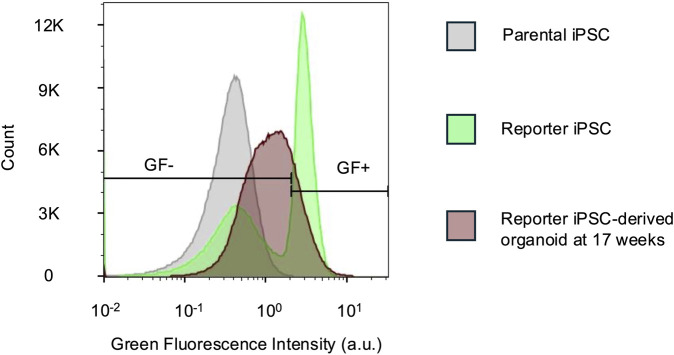
Unamplified native reporter fluorescence is detectable by flow cytometry. Flow cytometry histograms comparing green fluorescence (GF) intensities in parental and reporter iPSCs and dissociated 17-week-old reporter organoids. The iPSCs were used to determine the GF +/- gate. For the organoids, 78.7% cells were GF-. n = 444,565 cells for all samples. a.u. - arbitrary units.

For both the *UBE3A-YFP* mouse model (Dindot et al., 2008) and the imaging experiments in this study, antibody-based signal enhancement is required to visualize fluorescent protein expression. Flow cytometry provides a potential alternative measurement method with high sensitivity as well as single-cell resolution. Indeed, we observe that flow cytometry is able to distinguish unedited parental cells from cells edited with the reporter (Figure 4). Furthermore, 17-week-old cerebral organoids display intermediate fluorescence levels above the parental iPSC baseline and below the reporter iPSC signal (Figure 4). This reflects a decline in reporter fluorescence over time, consistent with *UBE3A* silencing. These results demonstrate that flow cytometry can reliably detect endogenous reporter fluorescence and its silencing trajectory, offering an antibody-independent method to monitor UBE3A dynamics.

### 3.5 Reporter fluorescence changes in response to topoisomerase inhibitors

**FIGURE 5:**
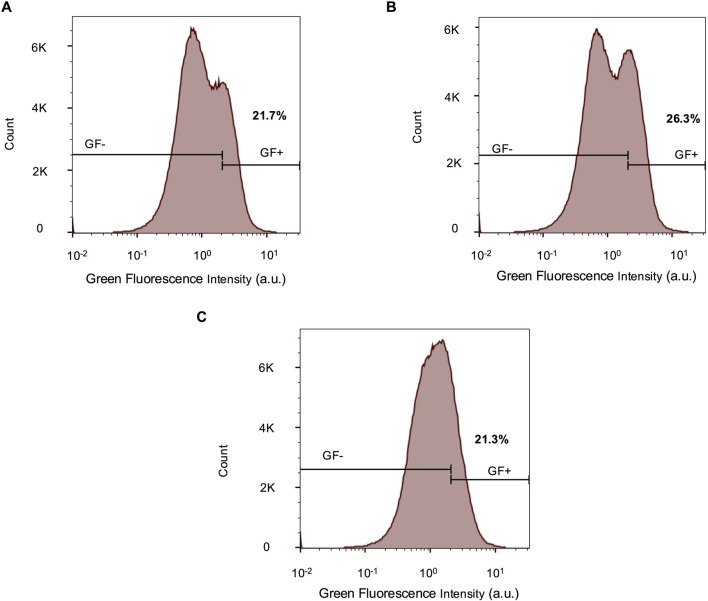
Reporter fluorescence changes in response to topoisomerase inhibitors. Flow cytometry histograms comparing green fluorescence intensities in 17-week-old reporter organoids exposed to **(A)** 1 µM topotecan, **(B)** 1 µM irinotecan, and **(C)** 0.2% DMSO in water (vehicle control). The parental and reporter iPSCs were used to determine the GF +/- gate. n = 444, 565 for all conditions. a.u. - arbitrary units.

Paternal *UBE3A*, which becomes epigenetically silenced in mature neurons, can be reactivated through various molecular strategies (Almeida et al., 2025). A potential application of the reporter cell line is to facilitate screening of novel compounds capable of inducing such reactivation. One of the first classes of molecules discovered to reactivate paternal *UBE3A* is topoisomerase inhibitors, specifically topotecan, which has previously reactivated *UBE3A* in human cerebral organoids (Sen et al., 2020), and irinotecan, which demonstrated similar effects in a *UBE3A-YFP* mouse model. They function by prematurely terminating the *UBE3A-ATS* transcript, thereby permitting expression of *UBE3A* (Huang et al., 2012). The expectation is that topotecan or irinotecan treatment of reporter organoids older than week 15, when both *UBE3A* and reporter fluorescence are largely silenced in neurons, would reactivate reporter fluorescence. We observe an interesting bifurcation of the cell population in 17-week-old organoids following either topotecan or irinotecan treatment. In the bimodal populations, one subpopulation exhibits increased reporter intensity, and the other subpopulation exhibits lower intensities compared to untreated organoids (Figures 5A–C). Flow cytometry gating based on parental (negative control) and reporter (positive control) iPSCs, indicate a higher proportion of reporter-positive cells in organoids treated with topoisomerase inhibitors and also a corresponding increase in mean fluorescence intensity (Supplementary Table S2).

### 3.6 Fusion of the reporter with UBE3A reduces enzyme activity

**FIGURE 6:**
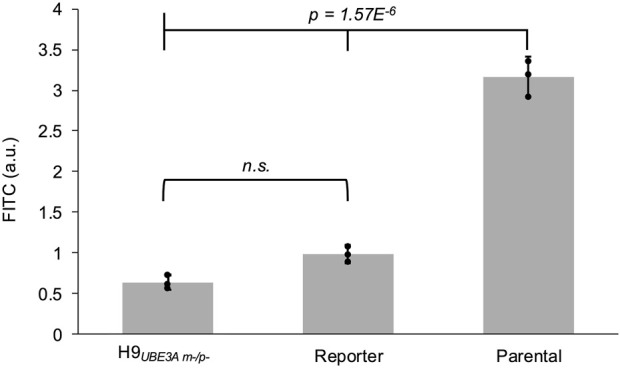
Fusion of the reporter with UBE3A reduces enzyme activity. Normalized fluorescence readings from the UBE3A activity assay performed on the cell lysates of H9_*UBE3A*__*m-/p-*_ ESCs, reporter iPSCs, and parental iPSCs. Error bars represent 95% confidence intervals. Black circles represent each biological replicate (3 per cell line). Full tick mark compared with half tick marks using one-way ANOVA followed by Tukey-Kramer *post hoc* test. a.u. - arbitrary units, n.s. - not significant.

Fusion of mGL with UBE3A via the GS Linker, while enabling reporting of its transcriptional activation, could affect the enzymatic activity of UBE3A. UBE3A functions as an E3 ligase in the ubiquitin proteasome system (Khatri and Man, 2019). To test this, UBE3A ubiquitin ligase activity in the reporter iPSCs can be measured using a recently developed ubiquitin conjugation and pull-down assay (Han et al., 2025). Using this assay, we observe the ubiquitin ligase activity of the reporter iPSCs is significantly reduced compared to that of the parental iPSCs. H9_*UBE3A*__*m-/p-*_ ESCs, in which *UBE3A* was deleted, were used as background control for non-specific activity (Figure 6).

## 4 Discussion

The reporter cell line presents both advantages and disadvantages. The utility of this model relies on genomic stability and reporter expression over long differentiation timelines. Previous studies with human brain organoid cultures have demonstrated genomic stability for at least 30 days (Navarro et al., 2020), and maintenance of neurodevelopmental and epigenetic signatures consistent with human fetal samples for at least 100 days (Luo et al., 2016). In this study, reporter expression decreases in TUJ1+ neurons due to *UBE3A* silencing as expected (Stanurova et al., 2016; Hsiao et al., 2019) and cannot be used to evaluate the stability of the reporter in organoids. However, *UBE3A* does not silence in SOX2+ cells and maintains stable reporter intensities for at least 12 weeks in culture. Furthermore, comparison of reporter and parental organoids at 18 weeks demonstrates that GFP fluorescence persists and remains clearly distinguishable from background signal (Supplementary Figure S7).

Prior correspondence with researchers in the field working in both mouse and human systems noted issues detecting fluorescence signals with direct fusions of fluorescence proteins to UBE3A, potentially due to the misfolding of the fluorescent protein. This therefore necessitates the use of fixation and antibody labeling of the fluorescent protein (Dindot et al., 2008; Lee et al., 2018; Huang et al., 2012; Meng et al., 2015; Lee et al., 2023; Clarke et al., 2024; Schmid et al., 2021; Vihma et al., 2024; Judson et al., 2014; McCoy et al., 2017; Jones et al., 2016; Condon et al., 2013; Hillman et al., 2017). Direct fusions to UBE3A in human cell lines seem to face issues of detection even with antibody labeling (Chen, 2016). The compatibility of this cell line with antibody-free detection using flow cytometry would support accelerated screening of novel compound libraries. The single-cell resolution of flow cytometry could also be leveraged to include cell-type-specific antibodies and to query reporter responses in individual cell types.

However, a key limitation of the reporter cell line is the ablation of UBE3A’s E3 ligase activity. We found that this enzymatic activity is not significantly higher compared to the H9_*UBE3A*__*m-/p-*_ cell line where UBE3A is deleted (Sirois, 2018; Sirois et al., 2020; Fink et al., 2017). This enzymatic function is essential for regulating cell cycle progression, neurodevelopment, and synaptic maturation in human cells (Khatri and Man, 2019; Estridge et al., 2025). While the reporter cells can identify novel UBE3A reactivators, the absence of E3 ligase activity hinders the assessment of downstream phenotypic recovery such as improved synaptic plasticity, dendrite formation, and electrophysiological function (Biagioni et al., 2024; Khatri and Man, 2019; Sen et al., 2020). Moreover, precise dosing of functional UBE3A is critical for phenotypic rescue, as overexpression has been associated with autism-like phenotypes (Khatri and Man, 2019). Novel reactivation candidates identified using the reporter model can be investigated for UBE3A enzymatic activity using the unedited parental cell line along with other AS iPSC lines. It is also important to note that the scope of use for this reporter is limited to AS maternal deletion etiologies.

Upon treatment of reporter organoids with topotecan and irinotecan, we observe two subpopulations. One subpopulation exhibits an increase in reporter intensity over the untreated organoids. The number of cells with an increase in intensity was modest, which is important to note as cerebral organoids are inherently heterogeneous. In addition, there was the emergence of a lower intensity subpopulation. Interestingly, immunofluorescence studies for topotecan exposed organoids also show a bimodal reporter expression, along with slightly increased UBE3A intensity over the vehicle control organoids (Supplementary Figure S8). While direct binding of topotecan and irinotecan with eGFP and eGFP-derivatives has not been reported, it is possible that they interact with these fluorescent proteins via other intracellular proteins to dampen fluorescence. Furthermore, topoisomerase inhibitors function by inducing double-stranded DNA breaks (Lee et al., 2018). While the doses used in this study are not expected to be toxic, DNA damage-induced stress responses might still be triggered. This shift in cellular stress can lead to pleiotropic effects influencing the expression of other genes, including those that might directly downregulate UBE3A. Moreover, stress phenotypes like elevated reactive oxygen species (ROS) levels has been shown to reduce GFP fluorescence (Ansari et al., 2016; Greenbaum et al., 2000).

## 5 Conclusion

Overall, this study develops a human-specific paternal *UBE3A* fluorescent reporter cell line. This model can be used for investigating the mechanism of UBE3A in driving AS phenotypes in neurodevelopmental cell types. Moreover, this reporter system could be a useful preclinical tool to screen therapeutic candidates targeting paternal *UBE3A* reactivation.

## Data Availability

The original contributions presented in the study are included in the article/Supplementary Material, further inquiries can be directed to the corresponding author.
