# Supplementary material for: A human Angelman Syndrome class II pluripotent stem cell line with fluorescent paternal UBE3A reporter
Source: Front Cell Dev Biol. 2025 Aug 29;13:1665693. doi: 10.3389/fcell.2025.1665693 (PMC12426290; doi:10.3389/fcell.2025.1665693)
Supplement: Supplementary file 1 [file DataSheet2.pdf]

```
CellProfiler Pipeline: http://www.cellprofiler.org
Version:5
DateRevision:426
GitHash:
ModuleCount:7
HasImagePlaneDetails:False

Images:[module_num:1|svn_version:'Unknown'|variable_revision_number:2|
show_window:False|notes:['To begin creating your project, use the
Images module to compile a list of files and/or folders that you want
to analyze. You can also specify a set of rules to include only the
desired files in your selected folders.']|batch_state:array([],
dtype=uint8)|enabled:True|wants_pause:False]
    :
    Filter images?:Images only
    Select the rule criteria:and (extension does isimage) (directory
doesnot containregexp "[\\\\/]\\.")

Metadata:[module_num:2|svn_version:'Unknown'|
variable_revision_number:6|show_window:False|notes:['The Metadata
module optionally allows you to extract information describing your
images (i.e, metadata) which will be stored along with your
measurements. This information can be contained in the file name and/
or location, or in an external file.']|batch_state:array([],
dtype=uint8)|enabled:True|wants_pause:False]
    Extract metadata?:No
    Metadata data type:Text
    Metadata types:{}
    Extraction method count:1
    Metadata extraction method:Extract from file/folder names
    Metadata source:File name
    Regular expression to extract from file name:^(?P<Plate>.*)_(?
P<Well>[A-P][0-9]{2})_s(?P<Site>[0-9])_w(?P<ChannelNumber>[0-9])
    Regular expression to extract from folder name:(?P<Date>[0-9]{4}
_[0-9]{2}_[0-9]{2})$
    Extract metadata from:All images
    Select the filtering criteria:and (file does contain "")
    Metadata file location:Elsewhere...|
    Match file and image metadata:[]
    Use case insensitive matching?:No
    Metadata file name:None
    Does cached metadata exist?:No

NamesAndTypes:[module_num:3|svn_version:'Unknown'|
variable_revision_number:8|show_window:False|notes:['The NamesAndTypes
module allows you to assign a meaningful name to each image by which
other modules will refer to it.']|batch_state:array([], dtype=uint8)|
enabled:True|wants_pause:False]
    Assign a name to:All images
    Select the image type:Color image
```

```
    Name to assign these images:Rep
    Match metadata:[]
    Image set matching method:Order
    Set intensity range from:Image metadata
    Assignments count:1
    Single images count:0
    Maximum intensity:255.0
    Process as 3D?:No
    Relative pixel spacing in X:1.0
    Relative pixel spacing in Y:1.0
    Relative pixel spacing in Z:1.0
    Select the rule criteria:and (file does contain "")
    Name to assign these images:DNA
    Name to assign these objects:Cell
    Select the image type:Grayscale image
    Set intensity range from:Image metadata
    Maximum intensity:255.0

Groups:[module_num:4|svn_version:'Unknown'|variable_revision_number:2|
show_window:False|notes:['The Groups module optionally allows you to
split your list of images into image subsets (groups) which will be
processed independently of each other. Examples of groupings include
screening batches, microtiter plates, time-lapse movies, etc.']|
batch_state:array([], dtype=uint8)|enabled:True|wants_pause:False]
    Do you want to group your images?:No
    grouping metadata count:1
    Metadata category:None

ColorToGray:[module_num:5|svn_version:'Unknown'|
variable_revision_number:4|show_window:False|notes:[]|
batch_state:array([], dtype=uint8)|enabled:True|wants_pause:False]
    Select the input image:Rep
    Conversion method:Split
    Image type:Channels
    Name the output image:OrigGray
    Relative weight of the red channel:1.0
    Relative weight of the green channel:1.0
    Relative weight of the blue channel:1.0
    Convert red to gray?:Yes
    Name the output image:OrigRed
    Convert green to gray?:Yes
    Name the output image:OrigGreen
    Convert blue to gray?:Yes
    Name the output image:OrigBlue
    Convert hue to gray?:Yes
    Name the output image:OrigHue
    Convert saturation to gray?:Yes
    Name the output image:OrigSaturation
    Convert value to gray?:Yes
    Name the output image:OrigValue
```

```
    Channel count:4
    Channel number:1
    Relative weight of the channel:1.0
    Image name:Blue
    Channel number:2
    Relative weight of the channel:1.0
    Image name:Green
    Channel number:3
    Relative weight of the channel:1.0
    Image name:Red
    Channel number:4
    Relative weight of the channel:1.0
    Image name:Magenta

MeasureColocalization:[module_num:6|svn_version:'Unknown'|
variable_revision_number:5|show_window:False|notes:[]|
batch_state:array([], dtype=uint8)|enabled:True|wants_pause:False]
    Select images to measure:Green, Magenta
    Set threshold as percentage of maximum intensity for the
images:15.0
    Select where to measure correlation:Across entire image
    Select objects to measure:
    Run all metrics?:No
    Calculate correlation and slope metrics?:Yes
    Calculate the Manders coefficients?:Yes
    Calculate the Rank Weighted Colocalization coefficients?:Yes
    Calculate the Overlap coefficients?:Yes
    Calculate the Manders coefficients using Costes auto
threshold?:Yes
    Method for Costes thresholding:Accurate

ExportToSpreadsheet:[module_num:7|svn_version:'Unknown'|
variable_revision_number:13|show_window:False|notes:[]|
batch_state:array([], dtype=uint8)|enabled:True|wants_pause:False]
    Select the column delimiter:Comma (",")
    Add image metadata columns to your object data file?:No
    Add image file and folder names to your object data file?:No
    Select the measurements to export:No
    Calculate the per-image mean values for object measurements?:No
    Calculate the per-image median values for object measurements?:No
    Calculate the per-image standard deviation values for object
measurements?:No
    Output file location:Default Output Folder|
    Create a GenePattern GCT file?:No
    Select source of sample row name:Metadata
    Select the image to use as the identifier:None
    Select the metadata to use as the identifier:None
    Export all measurement types?:No
    Press button to select measurements:
    Representation of Nan/Inf:NaN
```

```
    Add a prefix to file names?:No
    Filename prefix:
    Overwrite existing files without warning?:No
    Data to export:Image
    Combine these object measurements with those of the previous object?:No
    File name:Overlap-UBE3A+GFP-New-NoManders-2A.csv
    Use the object name for the file name?:No
```
