# Supplementary figures and images for: A human Angelman Syndrome class II pluripotent stem cell line with fluorescent paternal UBE3A reporter
Source: Front Cell Dev Biol. 2025 Aug 29;13:1665693. doi: 10.3389/fcell.2025.1665693 (PMC12426290; doi:10.3389/fcell.2025.1665693)

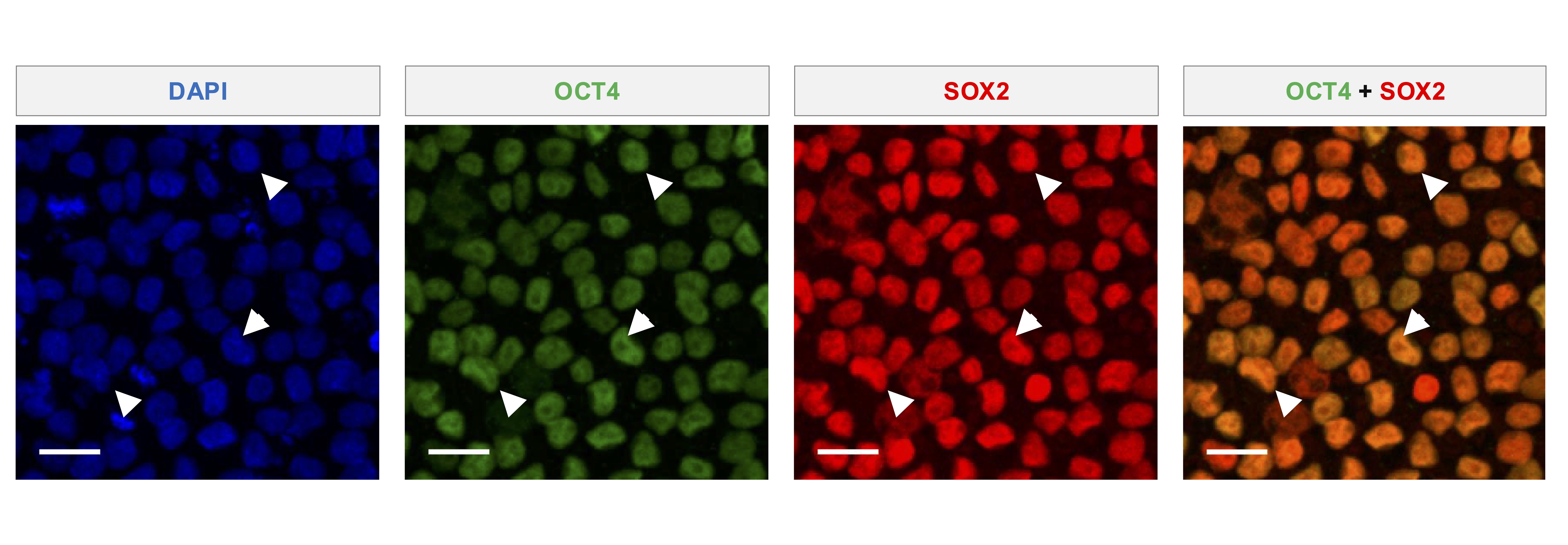

Supplement: Supplementary file 3 [file Image3.jpeg]

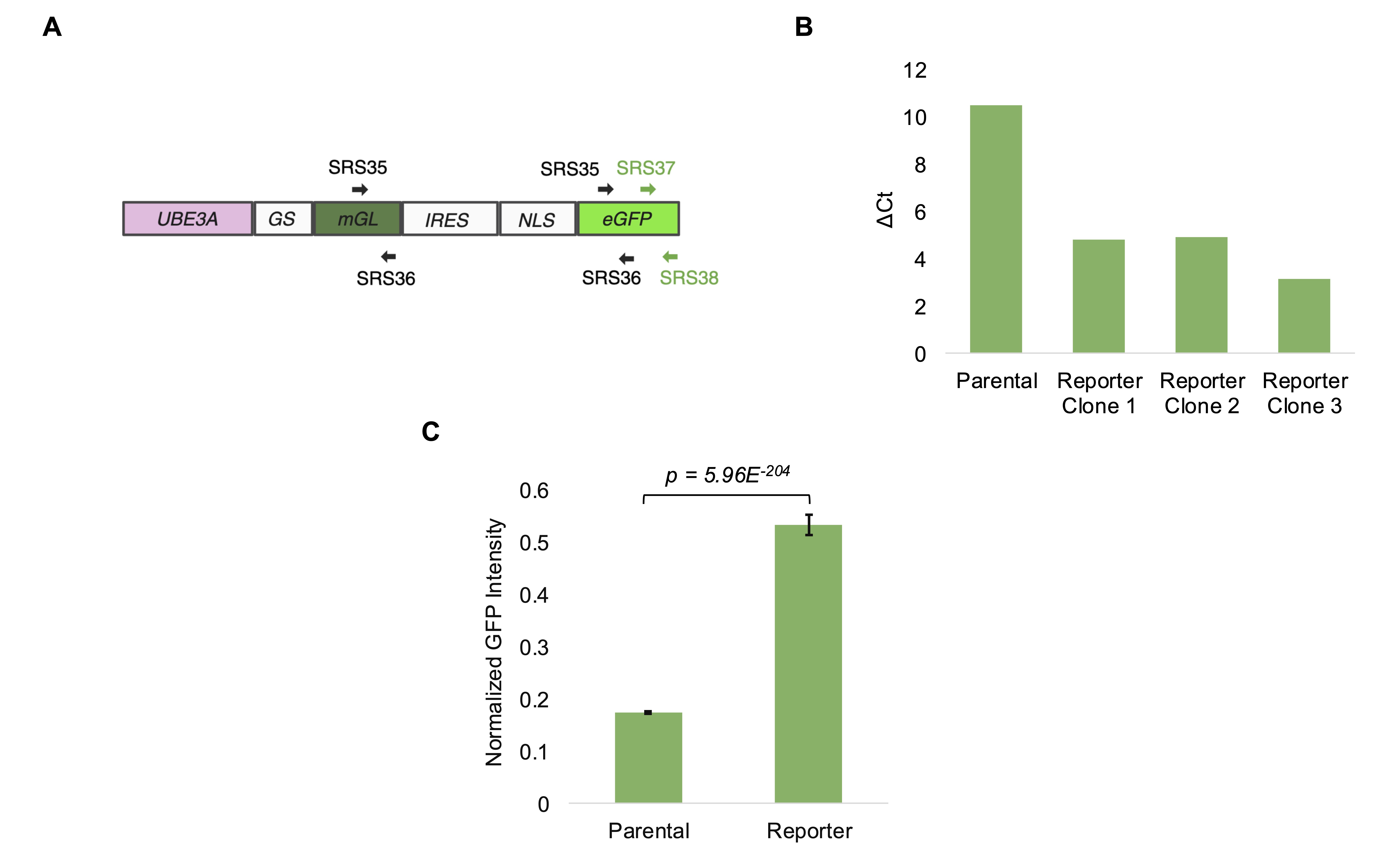

Supplement: Supplementary file 6 [file Image1.jpeg]

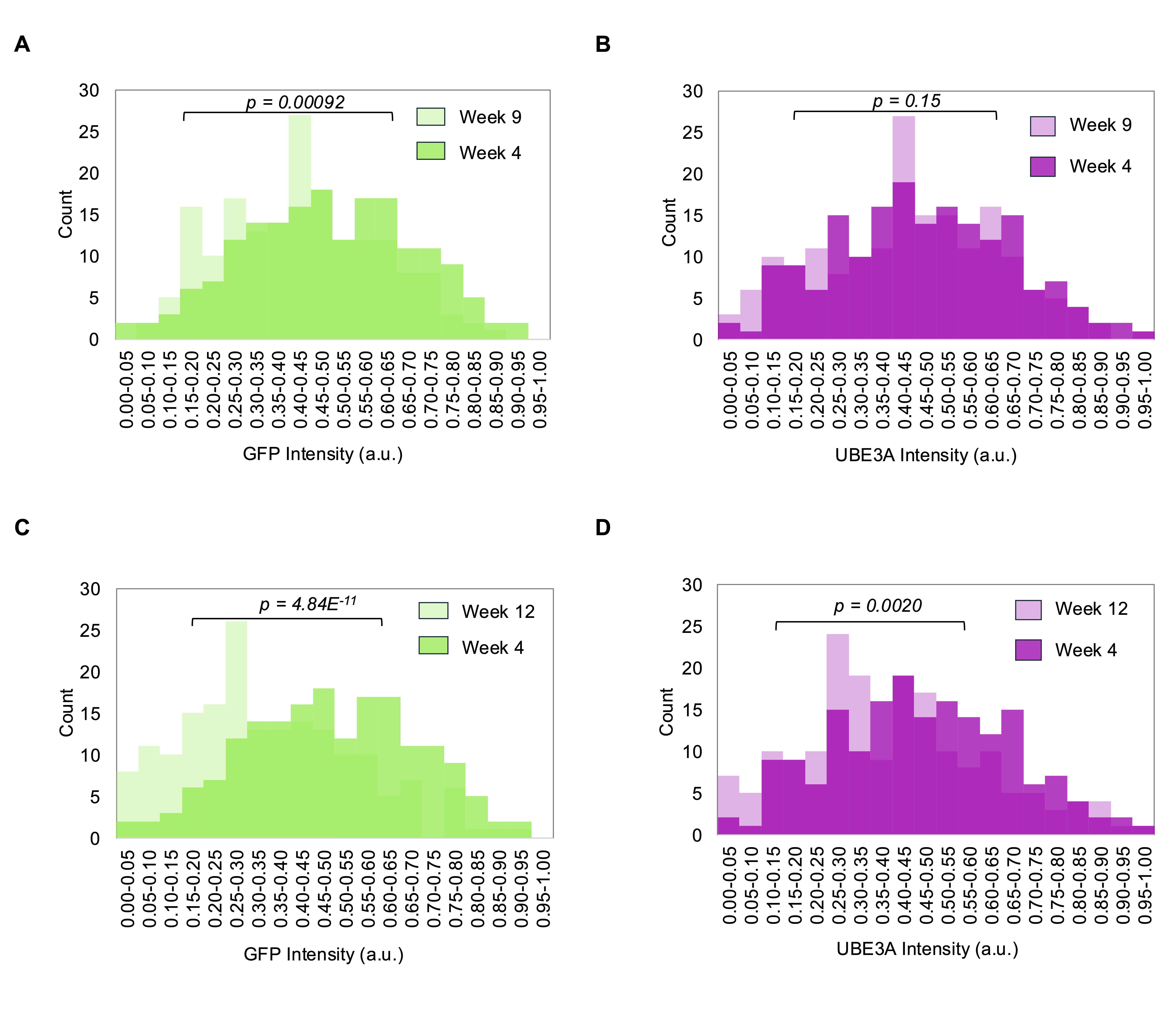

Supplement: Supplementary file 7 [file Image4.jpeg]

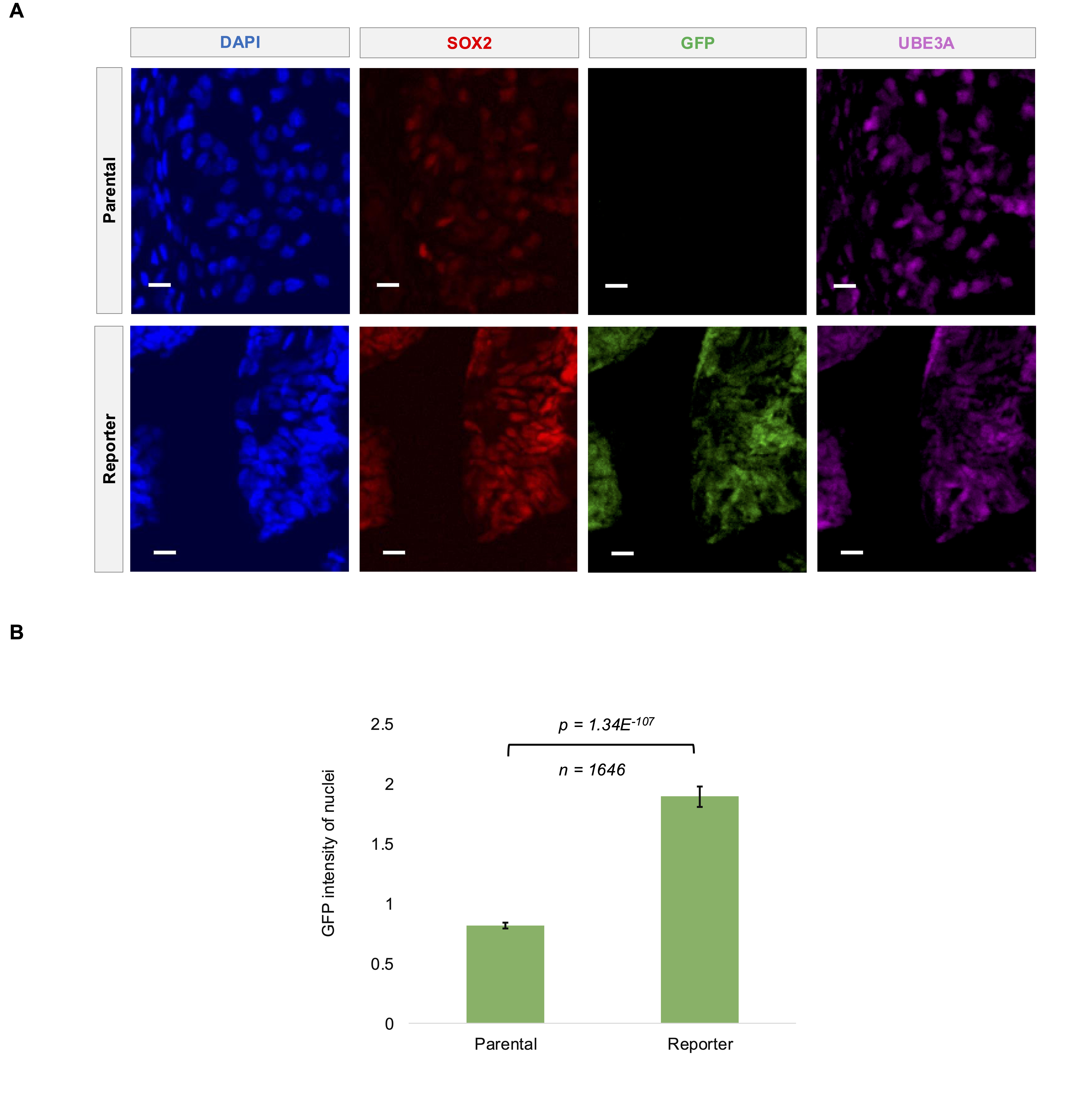

Supplement: Supplementary file 8 [file Image7.jpeg]

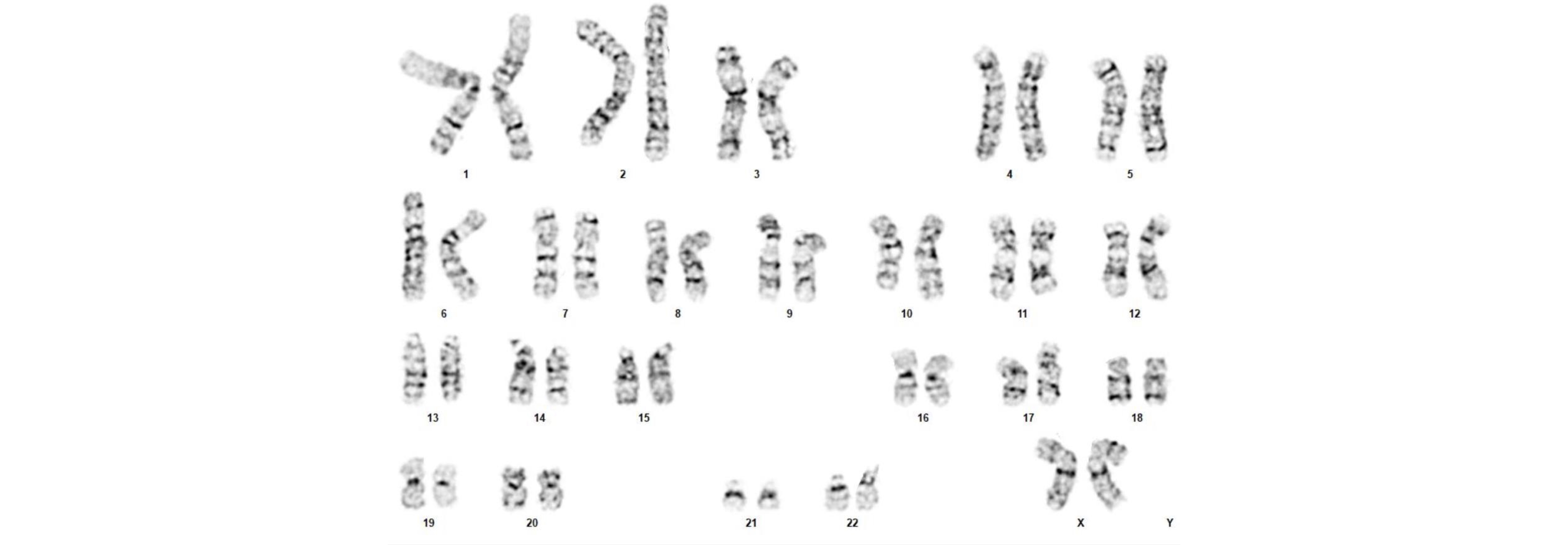

Supplement: Supplementary file 9 [file Image2.jpeg]

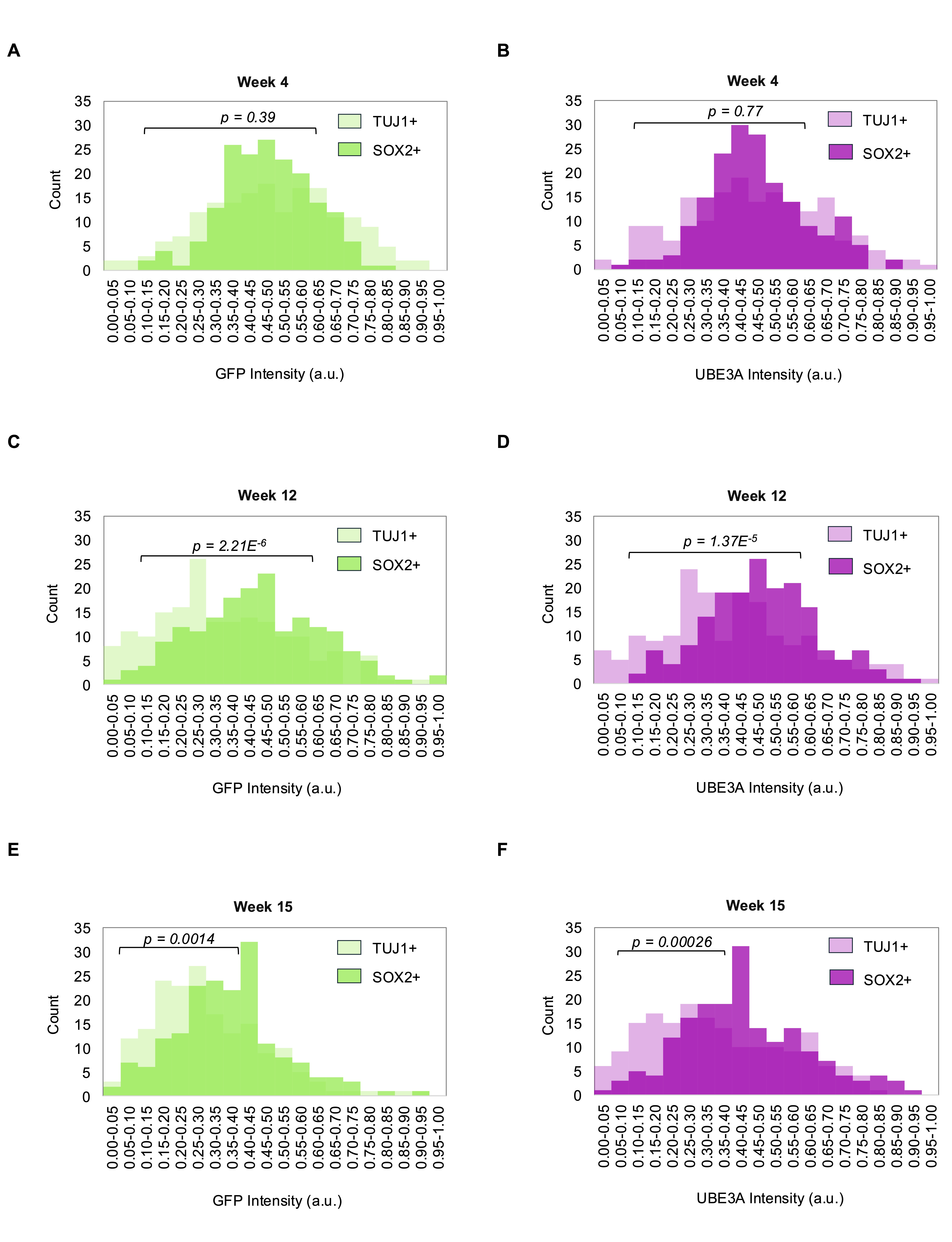

Supplement: Supplementary file 10 [file Image5.jpeg]

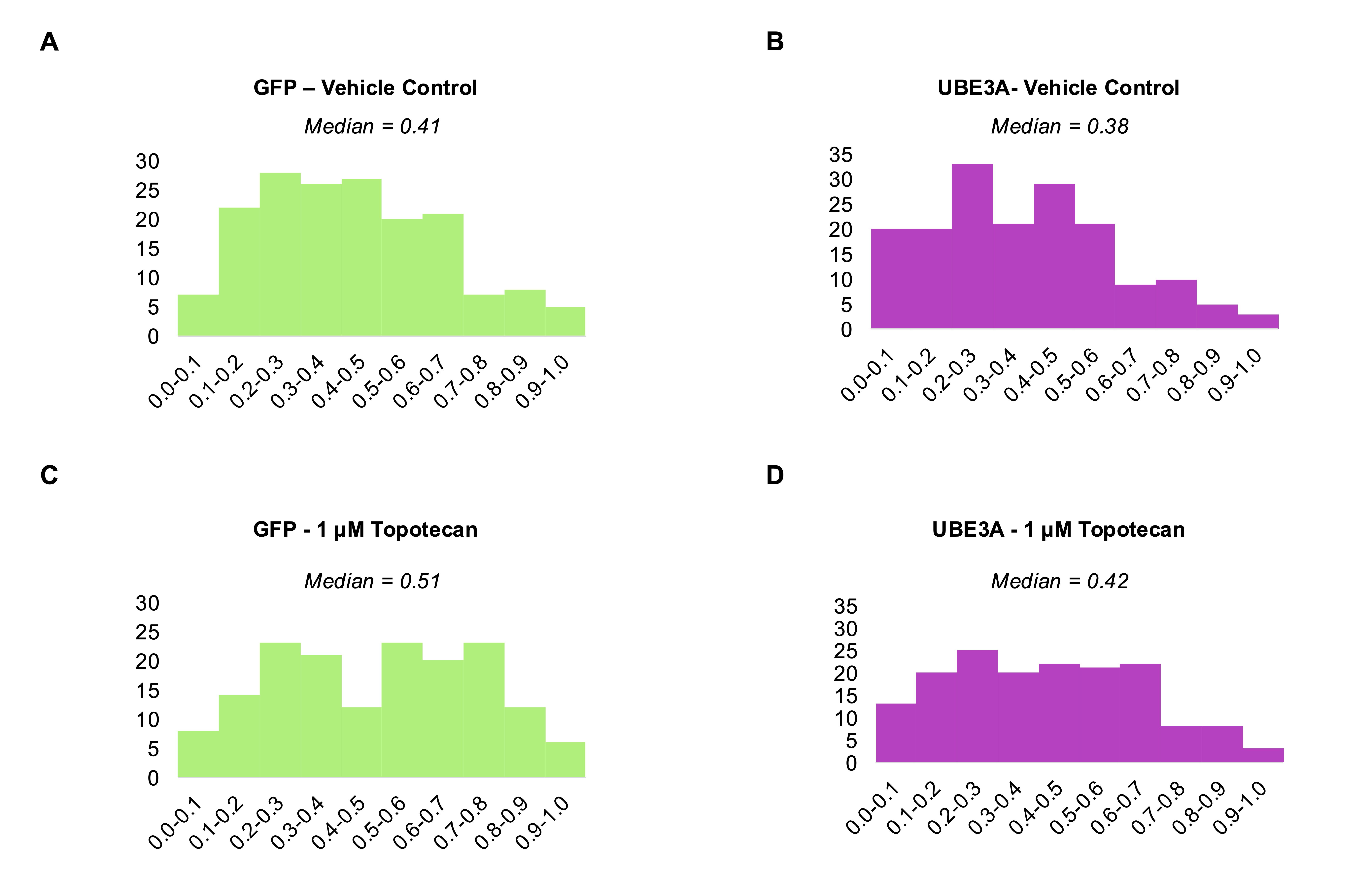

Supplement: Supplementary file 14 [file Image8.jpeg]

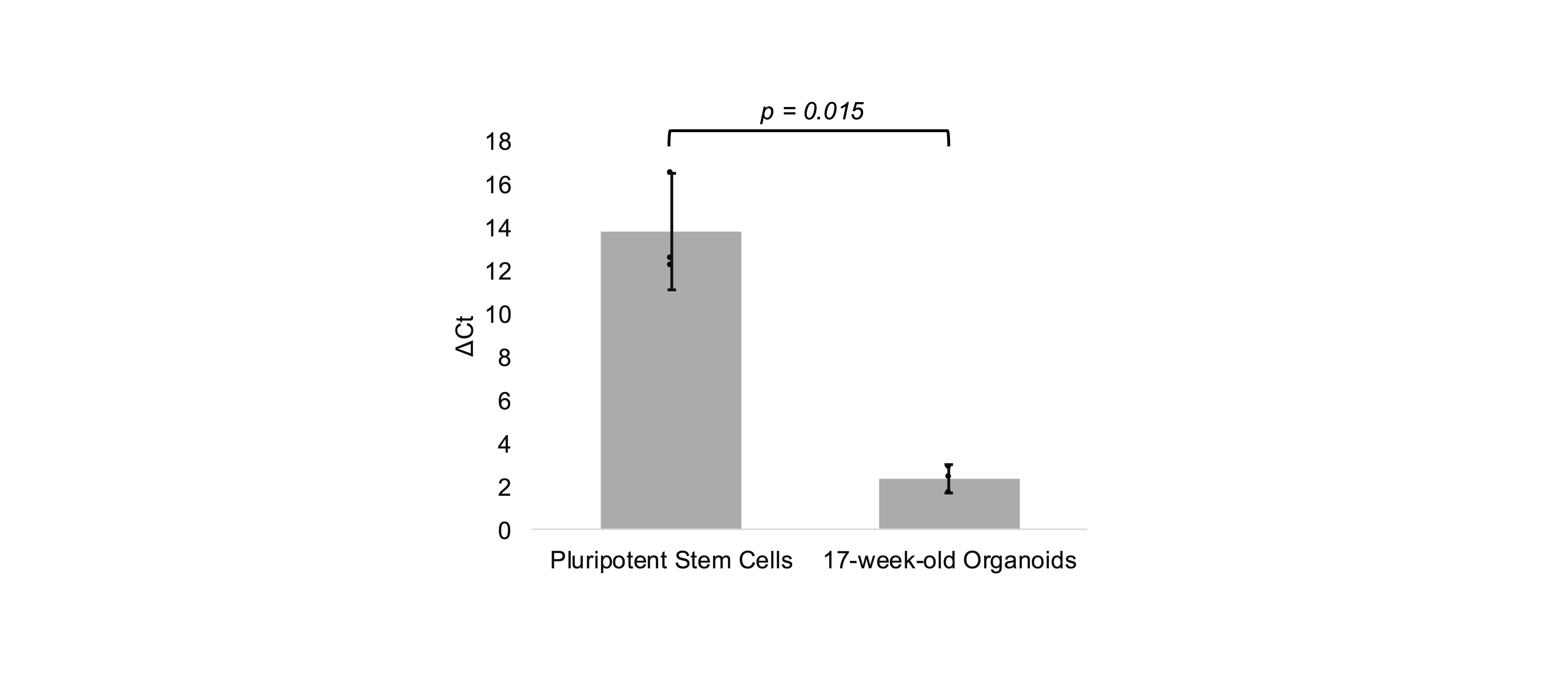

Supplement: Supplementary file 16 [file Image6.jpeg]
