# Supplementary material for: A human Angelman Syndrome class II pluripotent stem cell line with fluorescent paternal UBE3A reporter
Source: Front Cell Dev Biol. 2025 Aug 29;13:1665693. doi: 10.3389/fcell.2025.1665693 (PMC12426290; doi:10.3389/fcell.2025.1665693)
Supplement: Supplementary file 4 [file DataSheet4.pdf]

```
CellProfiler Pipeline: http://www.cellprofiler.org
Version:5
DateRevision:426
GitHash:
ModuleCount:8
HasImagePlaneDetails:False

Images:[module_num:1|svn_version:'Unknown'|variable_revision_number:2|
show_window:False|notes:['To begin creating your project, use the
Images module to compile a list of files and/or folders that you want
to analyze. You can also specify a set of rules to include only the
desired files in your selected folders.']|batch_state:array([],
dtype=uint8)|enabled:True|wants_pause:False]
    :
    Filter images?:Images only
    Select the rule criteria:and (extension does isimage) (directory
doesnot containregexp "[\\\\/]\\.")

Metadata:[module_num:2|svn_version:'Unknown'|
variable_revision_number:6|show_window:False|notes:['The Metadata
module optionally allows you to extract information describing your
images (i.e, metadata) which will be stored along with your
measurements. This information can be contained in the file name and/
or location, or in an external file.']|batch_state:array([],
dtype=uint8)|enabled:True|wants_pause:False]
    Extract metadata?:No
    Metadata data type:Text
    Metadata types:{}
    Extraction method count:1
    Metadata extraction method:Extract from file/folder names
    Metadata source:File name
    Regular expression to extract from file name:^(?P<Plate>.*)_(?
P<Well>[A-P][0-9]{2})_s(?P<Site>[0-9])_w(?P<ChannelNumber>[0-9])
    Regular expression to extract from folder name:(?P<Date>[0-9]{4}
_[0-9]{2}_[0-9]{2})$
    Extract metadata from:All images
    Select the filtering criteria:and (file does contain "")
    Metadata file location:Elsewhere...|
    Match file and image metadata:[]
    Use case insensitive matching?:No
    Metadata file name:None
    Does cached metadata exist?:No

NamesAndTypes:[module_num:3|svn_version:'Unknown'|
variable_revision_number:8|show_window:False|notes:['The NamesAndTypes
module allows you to assign a meaningful name to each image by which
other modules will refer to it.']|batch_state:array([], dtype=uint8)|
enabled:True|wants_pause:False]
    Assign a name to:Images matching rules
    Select the image type:Color image
```

```
    Name to assign these images:BT-
    Match metadata:[]
    Image set matching method:Order
    Set intensity range from:Image metadata
    Assignments count:1
    Single images count:0
    Maximum intensity:255.0
    Process as 3D?:No
    Relative pixel spacing in X:1.0
    Relative pixel spacing in Y:1.0
    Relative pixel spacing in Z:1.0
    Select the rule criteria:and (file does contain "MAX")
    Name to assign these images:A3StemCells
    Name to assign these objects:Cell
    Select the image type:Color image
    Set intensity range from:Image metadata
    Maximum intensity:255.0

Groups:[module_num:4|svn_version:'Unknown'|variable_revision_number:2|
show_window:False|notes:['The Groups module optionally allows you to
split your list of images into image subsets (groups) which will be
processed independently of each other. Examples of groupings include
screening batches, microtiter plates, time-lapse movies, etc.']|
batch_state:array([], dtype=uint8)|enabled:True|wants_pause:False]
    Do you want to group your images?:No
    grouping metadata count:1
    Metadata category:None

ColorToGray:[module_num:5|svn_version:'Unknown'|
variable_revision_number:4|show_window:False|notes:[]|
batch_state:array([], dtype=uint8)|enabled:True|wants_pause:False]
    Select the input image:A3StemCells
    Conversion method:Split
    Image type:Channels
    Name the output image:OrigGray
    Relative weight of the red channel:1.0
    Relative weight of the green channel:1.0
    Relative weight of the blue channel:1.0
    Convert red to gray?:Yes
    Name the output image:OrigRed
    Convert green to gray?:Yes
    Name the output image:OrigGreen
    Convert blue to gray?:Yes
    Name the output image:OrigBlue
    Convert hue to gray?:Yes
    Name the output image:OrigHue
    Convert saturation to gray?:Yes
    Name the output image:OrigSaturation
    Convert value to gray?:Yes
    Name the output image:OrigValue
```

```
    Channel count:2
    Channel number:1
    Relative weight of the channel:1.0
    Image name:Blue
    Channel number:2
    Relative weight of the channel:1.0
    Image name:Green

IdentifyPrimaryObjects:[module_num:6|svn_version:'Unknown'|
variable_revision_number:15|show_window:False|notes:[]|
batch_state:array([], dtype=uint8)|enabled:True|wants_pause:False]
    Select the input image:Blue
    Name the primary objects to be identified:DAPICells
    Typical diameter of objects, in pixel units (Min,Max):5,50
    Discard objects outside the diameter range?:Yes
    Discard objects touching the border of the image?:No
    Method to distinguish clumped objects:Intensity
    Method to draw dividing lines between clumped objects:Intensity
    Size of smoothing filter:4
    Suppress local maxima that are closer than this minimum allowed
distance:3
    Speed up by using lower-resolution image to find local maxima?:No
    Fill holes in identified objects?:After both thresholding and
declumping
    Automatically calculate size of smoothing filter for
declumping?:No
    Automatically calculate minimum allowed distance between local
maxima?:No
    Handling of objects if excessive number of objects
identified:Continue
    Maximum number of objects:500
    Use advanced settings?:Yes
    Threshold setting version:12
    Threshold strategy:Adaptive
    Thresholding method:Minimum Cross-Entropy
    Threshold smoothing scale:2
    Threshold correction factor:0.7
    Lower and upper bounds on threshold:0.0,1.0
    Manual threshold:0.0
    Select the measurement to threshold with:None
    Two-class or three-class thresholding?:Three classes
    Log transform before thresholding?:No
    Assign pixels in the middle intensity class to the foreground or
the background?:Background
    Size of adaptive window:30
    Lower outlier fraction:0.05
    Upper outlier fraction:0.05
    Averaging method:Mean
    Variance method:Standard deviation
    # of deviations:2.0
```

```
    Thresholding method:Otsu

MeasureObjectIntensity:[module_num:7|svn_version:'Unknown'|
variable_revision_number:4|show_window:False|notes:[]|
batch_state:array([], dtype=uint8)|enabled:True|wants_pause:False]
    Select images to measure:Blue, Green
    Select objects to measure:DAPICells

ExportToSpreadsheet:[module_num:8|svn_version:'Unknown'|
variable_revision_number:13|show_window:False|notes:[]|
batch_state:array([], dtype=uint8)|enabled:True|wants_pause:False]
    Select the column delimiter:Comma (",")
    Add image metadata columns to your object data file?:No
    Add image file and folder names to your object data file?:Yes
    Select the measurements to export:No
    Calculate the per-image mean values for object measurements?:No
    Calculate the per-image median values for object measurements?:No
    Calculate the per-image standard deviation values for object
measurements?:No
    Output file location:Default Output Folder|
    Create a GenePattern GCT file?:No
    Select source of sample row name:Metadata
    Select the image to use as the identifier:None
    Select the metadata to use as the identifier:None
    Export all measurement types?:No
    Press button to select measurements:DAPICells|
Intensity_MinIntensity_Green,DAPICells|
Intensity_MedianIntensity_Green,DAPICells|
Intensity_IntegratedIntensity_Green,DAPICells|
Intensity_StdIntensity_Green,DAPICells|
Intensity_MeanIntensityEdge_Green,DAPICells|
Intensity_LowerQuartileIntensity_Green,DAPICells|
Intensity_MeanIntensity_Green,DAPICells|
Intensity_MaxIntensityEdge_Green,DAPICells|
Intensity_MinIntensityEdge_Green,DAPICells|
Intensity_StdIntensityEdge_Green,DAPICells|
Intensity_UpperQuartileIntensity_Green,DAPICells|
Intensity_MADIntensity_Green,DAPICells|
Intensity_IntegratedIntensityEdge_Green,DAPICells|
Intensity_MassDisplacement_Green,DAPICells|
Intensity_MaxIntensity_Green,DAPICells|
Location_CenterMassIntensity_Y_Green,DAPICells|
Location_CenterMassIntensity_Z_Green,DAPICells|
Location_CenterMassIntensity_X_Green,DAPICells|
Location_Center_X,DAPICells|Location_Center_Y,DAPICells|
Location_Center_Z,DAPICells|Location_MaxIntensity_Y_Green,DAPICells|
Location_MaxIntensity_Z_Green,DAPICells|
Location_MaxIntensity_X_Green,DAPICells|Number_Object_Number,Image|
URL_A3StemCells,Image|ModuleError_01Images,Image|
ModuleError_07MeasureObjectIntensity,Image|ModuleError_04Groups,Image|
```

```
ModuleError_06IdentifyPrimaryObjects,Image|
ModuleError_02Metadata,Image|ModuleError_05ColorToGray,Image|
ModuleError_03NamesAndTypes,Image|
Threshold_SumOfEntropies_DAPICells,Image|
Threshold_WeightedVariance_DAPICells,Image|
Threshold_OrigThreshold_DAPICells,Image|
Threshold_FinalThreshold_DAPICells,Image|
Threshold_GuideThreshold_DAPICells,Image|ExecutionTime_01Images,Image|
ExecutionTime_05ColorToGray,Image|ExecutionTime_02Metadata,Image|
ExecutionTime_03NamesAndTypes,Image|
ExecutionTime_06IdentifyPrimaryObjects,Image|
ExecutionTime_04Groups,Image|
ExecutionTime_07MeasureObjectIntensity,Image|
PathName_A3StemCells,Image|Group_Index,Image|Group_Length,Image|
Group_Number,Image|Count_DAPICells,Image|Height_A3StemCells,Image|
Frame_A3StemCells,Image|FileName_A3StemCells,Image|
Width_A3StemCells,Image|MD5Digest_A3StemCells,Image|
Scaling_A3StemCells,Image|Series_A3StemCells,Experiment|
Run_Timestamp,Experiment|CellProfiler_Version,Experiment|
Modification_Timestamp,Experiment|Pipeline_Pipeline
    Representation of Nan/Inf:Null
    Add a prefix to file names?:No
    Filename prefix:MyExpt_
    Overwrite existing files without warning?:No
    Data to export:DAPICells
    Combine these object measurements with those of the previous
object?:No
    File name:A3vsASDWeek18Intensity-NEW.csv
    Use the object name for the file name?:No
```
