# Supplementary material for: A human Angelman Syndrome class II pluripotent stem cell line with fluorescent paternal UBE3A reporter
Source: Front Cell Dev Biol. 2025 Aug 29;13:1665693. doi: 10.3389/fcell.2025.1665693 (PMC12426290; doi:10.3389/fcell.2025.1665693)
Supplement: Supplementary file 11 [file DataSheet5.docx]

**Description of Supplementary Material:**

**File name: Supplementary Data Sheet 1**

**Description:** All Supplementary Figures (with figure captions) and Supplementary Tables.

**File name: Supplementary Data Sheet 2**

**Description:** CellProfiler pipeline for Reporter and UBE3A overlap quantification in Figure 2A.

**File name: Supplementary Data Sheet 3**

**Description:** CellProfiler pipeline for Reporter intensity measurements reported in Supplementary Figure S1C.

**File name: Supplementary Data Sheet 4**

**Description:** CellProfiler pipeline for Reporter intensity measurements reported in Supplementary Figure S7B.

**File name: Supplementary Table 1**

**Description:** Raw data used to generate plots for Figures 1, 3, 6, Supplementary Figures S1, S4-S8.

**File name: Supplementary Table 2**

**Description:** Raw data used to generate plots for Figure 4.

**File name: Supplementary Table 3**

**Description:** Raw data used to generate plots for Figure 5.

**File name: Supplementary Image 1**

**Description:** Image file for Supplementary Figure S1.

**File name: Supplementary Image 2**

**Description:** Image file for Supplementary Figure S2.

**File name: Supplementary Image 3**

**Description:** Image file for Supplementary Figure S3.

**File name: Supplementary Image 4**

**Description:** Image file for Supplementary Figure S4.

**File name: Supplementary Image 5**

**Description:** Image file for Supplementary Figure S5.

**File name: Supplementary Image 6**

**Description:** Image file for Supplementary Figure S6.

**File name: Supplementary Image 7**

**Description:** Image file for Supplementary Figure S7.

**File name: Supplementary Image 8**

**Description:** Image file for Supplementary Figure S8.
