# Supplementary material for: A human Angelman Syndrome class II pluripotent stem cell line with fluorescent paternal UBE3A reporter
Source: Front Cell Dev Biol. 2025 Aug 29;13:1665693. doi: 10.3389/fcell.2025.1665693 (PMC12426290; doi:10.3389/fcell.2025.1665693)
Supplement: Supplementary file 15 [file DataSheet1.docx]

Supplementary Material

# Supplementary Figures and Tables

## Supplementary Figures

**Supplementary Figure S1.** RNA and protein level measurements to confirm reporter construct expression. A) qPCR Primer target regions within the transcript. Data from primers SRS35 and SRS36 (black) is shown in Figure 1C. B) Data for region amplified by primers SRS37 and SRS38. ∆Ct values relative to *GAPDH* presented for the parental iPSCs and the three monoclonal edited reporter iPSCs. C) Normalized GFP intensities for DAPI stained nuclei in Figure 1D. Reporter iPSCs show significantly higher GFP expression compared to the parental iPSCs. Samples were compared using a two-sample t-test (n=1560 for parental, n=1328 for reporter).

**Supplementary Figure S2.** G-banded chromosomes show normal chromosome counts of 46 XX for parental stem cells at passage number 43. The karyotype analysis was performed by LabCorp.

**Supplementary Figure S3.** Reporter iPSCs express pluripotency markers SOX2 (red) and OCT4 (green) post editing. Arrowheads point to representative nuclei (DAPI) expressing both markers. All scale bars are 10 µm.

**Supplementary Figure S4.** Intensity histograms from confocal images for nuclei in TUJ1+ cells, comparing A) GFP and B) UBE3A at week 4 to week 9, and C) GFP and D) UBE3A at week 4 to week 12. Populations were compared using a two-sample t-test, n=180 cells (3 organoids). a.u. – arbitrary units.

**Supplementary Figure S5.** GFP and UBE3A intensity histograms from confocal images for nuclei in TUJ1+ cells compared to SOX2+ nuclei at A) and B) week 4, C) and D) week 12, and E) and F) week 15. Populations were compared using a two-sample t-test, n = 180 cells (3 organoids). a.u.- arbitrary units.

**Supplementary Figure S6.** RT-qPCR measurements confirm increased UBE3A-ATS expression in 17-week-old reporter organoids compared to iPSCs. ∆Ct values relative to *PPIA* presented for the 17-week-old organoids and reporter iPSCs. Samples were compared using a two-sample t-test (n=3 biological replicates).

**Supplementary Figure S7.** Reporter expression is stable in long-term organoid cultures. A) Immunofluorescence images of 18-week-old whole brain organoids derived from reporter and parental stem cell lines. B) GFP intensity in SOX2+ nuclei of reporter-derived organoids is significantly higher compared to parental organoids. n = 1646. Populations were compared using a two-sample t-test. All scale bars are 10 µm.

**Supplementary Figure S8.** Reporter and UBE3A expression in 17-week-old organoids (n =3 each) exposed to A) and B) 0.2% DMSO in water as vehicle control and C) and D) 1 μM topotecan. n = 171 cells (control) and n = 162 cells (topotecan) were analyzed and presented.

## Supplementary Tables

**Table S1:** Primer Sequences.

| **Primer Name** | **Experiment** | **Sequence - 5' to 3'** |
| --- | --- | --- |
| SRS21 | Genomic PCR Screening – Round 1 and 2 | ACCTTGCATTCCTCGTCACA |
| SRS24 | Genomic PCR Screening – Round 1 | CCTCACATTGCCAAAAGACG |
| DSP206 | Genomic PCR Screening – Round 2 | CACTGATCACGTGCCTCGATA |
| SRS35 | qPCR: mGL-eGFP | AAGCAGAAGAACGGCATCAA |
| SRS36 | qPCR: mGL-eGFP | GGGGGTGTTCTGCTGGTAGT |
| SRS37 | qPCR: eGFP only | AGTCCGCCCTGAGCAAAGA |
| SRS38 | qPCR: eGFP only | TCCAGCAGGACCATGTGATC |

| **Sample** | **%GF+ Cells** | **For GF+ population** | | |
| --- | --- | --- | --- | --- |
|  |  | Geometric Mean | Median | Mode |
| Reporter Organoids + Vehicle Control | 21.3 | 3.02 | 2.78 | 2.11 |
| Reporter Organoids + 1μM Topotecan | 21.7 | 3.03 | 2.83 | 2.11 |
| Reporter Organoids + 1μM Irinotecan | 26.3 | 3.12 | 2.92 | 2.18 |
| Reporter iPSCs (Positive Control) | 50.9 | 3.07 | 2.99 | 2.80 |
| Parental iPSCs (Negative Control) | 0.12 | 2.81 | 2.50 | 2.11 |

**Supplementary Table S2:** Green fluorescence intensity (a.u.) data from flow cytometry experiments.
